# Visceral Leishmaniasis in Far Western Nepal: Another Case and Concerns about a New Area of Endemicity

**DOI:** 10.4269/ajtmh.2011.11-0021

**Published:** 2011-03-04

**Authors:** Dan Schwarz, Jason Andrews, Bikash Gauchan

**Affiliations:** Nyaya Health; Bayalpata Hospital; Ridikot, Achham, Nepal; Brown University School of Medicine; Providence, RI; Harvard School of Public Health; Boston, MA; E-mail: dan@nyayahealth.org; Nyaya Health; Bayalpata Hospital; Ridikot, Achham, Nepal; Brigham and Women's Hospital; Department of Medicine; Boston, MA; Nyaya Health; Bayalpata Hospital; Ridikot, Achham, Nepal

Dear Sir:

Pandey and others reported the first case of visceral leishmaniasis (VL) in the Far Western Hills region of Nepal.^1^ At our hospital in the Achham district, which neighbors the Doti district described in the Pandey and others report, we recently had a case of VL in a 17-year-old woman who presented with advanced disease. We transferred her to a hospital in Kathmandu where she received amphotericin and required intensive care, but unfortunately ultimately succumbed to her illness.

Like the patient Pandey and others described, our patient had never traveled to known VL-endemic areas. As the authors pointed out, VL has been thought to be limited to south-eastern Nepal, far from where these cases occurred. It is possible that these cases were due to *Leishmania infantum*, which has a canine reservoir and has been seen in Himachal Pradesh in India, Pakistan, and throughout Central Asia.^2^,^3^ However, it is also possible that the Phlebotomine sandfly is making inroads in other parts of Nepal, and with it *Leishmania donovani*. With climate change, we have seen other vector-borne diseases spreading to new areas. Dengue was first seen in southern Nepal in 2004 and has moved north to Kathmandu, where *Aedes aegypti* was not previously seen.^4^,^5^

Given extreme poverty and poor healthcare infrastructure in the remote western part of Nepal, passive case detection for VL may be inadequate for surveillance. To achieve the goal of VL eradication agreed upon by the governments of Nepal, India, and Bangladesh, we concur with Pandey and others that active surveillance in Nepal should be urgently expanded.
